# A metal-free salalen ligand with anti-tumor and synergistic activity in resistant leukemia and solid tumor cells via mitochondrial pathway

**DOI:** 10.1007/s00432-021-03679-3

**Published:** 2021-07-02

**Authors:** Sina M. Hopff, Qifang Wang, Corazon Frias, Marie Ahrweiler, Nicola Wilke, Nathalie Wilke, Albrecht Berkessel, Aram Prokop

**Affiliations:** 1 Department of Pediatric Hematology/Oncology, Municipal Clinics of Cologne, Children’s Hospital of the City Cologne, Amsterdamer Straße 59, 50735 Cologne, Germany; 2 Department of Chemistry, University of Cologne, Greinstraße 4, 50939 Cologne, Germany; 3 Department of Pediatric Hematology/Oncology, Helios Clinic Schwerin, Wismarsche Straße 393-397, 19055 Schwerin, Germany; 4 Medical School Hamburg (MSH), University of Applied Sciences and Medical University, Am Kaiserkai 1, 20457 Hamburg, Germany

**Keywords:** Metal-free ligand, Salalen, Mitochondrial pathway, Apoptosis, Synergistic effects, Multidrug resistance

## Abstract

**Purpose:**

Since the discovery of the well-known cis-platin, transition metal complexes are highly recognized as cytostatic agents. However, toxic side effects of the metal ions present in the complexes may pose significant problems for their future development. Therefore, we investigated the metal-free salalen ligand WQF 044.

**Methods:**

DNA fragmentations in leukemia (Nalm6) and solid tumor cells (BJAB, MelHO, MCF-7, RM82) proved the apoptotic effects of WQF 044, its overcoming of resistances and the cellular pathways that are affected by the substance. The apoptotic mechanisms finding were supported by western blot analysis, measurement of the mitochondrial membrane potential and polymerase chain reactions.

**Results:**

A complex intervention in the mitochondrial pathway of apoptosis with a Bcl-2 and caspase dependence was observed. Additionally, a wide range of tumors were affected by the ligand in a low micromolar range in-vitro. The compound overcame multidrug resistances in P-gp over-expressed acute lymphoblastic leukemia and CD95-downregulated Ewing’s sarcoma cells. Quite remarkable synergistic effects with vincristine were observed in Burkitt-like lymphoma cells.

**Conclusion:**

The investigation of a metal-free salalen ligand as a potential anti-cancer drug revealed in promising results for a future clinical use.

## Introduction

Developing new anti-cancer targets, especially against resistant tumors, is very challenging in cancer research (Kelland 2007). The discovery of the well-known cisplatin was pioneering on this field, but severe side effects limit the use of platin derivates (Bruijnincx and Sadler 2008; Kelland 2007; Rabik and Dolan 2007; Wild^2012^). Therefore finding new substances without a metal ion in its center is of high interest.

**Scheme 1:**
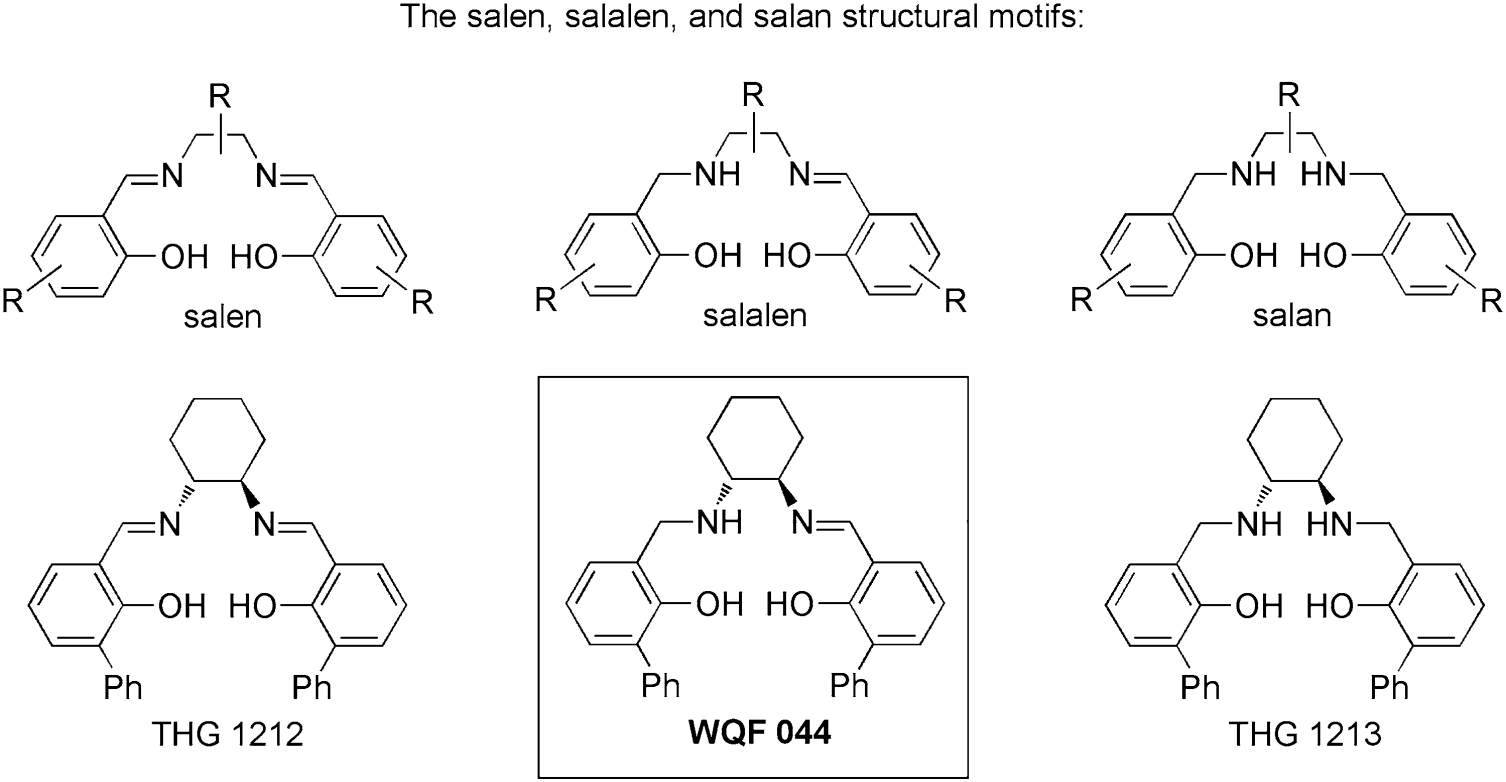
Top: General structures of the salen, the salalen, and the salan motifs. Bottom: Explicit structures of the salen THG 1212, the salalen WQF 044 (this work), and of the salan THG 1213

Salans, salalens and in particular salens are most prominent ligands in coordination chemistry, and many of their metal complexes have shown potent antitumor activity (Scheme 1, top) (Grutzke et al. 2015; Hopff et al. 2020; Immel et al. 2011, 2012a, b; Lee 2010, 2011; Meker et al. 2011; Mir et al. 2017; Peri et al. 2011; Terenzi^ 2016^). However, earlier work of ours on Mo-complexes had revealed that the biological activity may actually reside in the ligand. For example, the metal-free salan ligand THG 1213 (Scheme 1, bottom right) was in fact much more active than the corresponding Mo-complexes (Dragoun et al. 2018). Another striking observation was that the corresponding salen ligand THG 1212 (Scheme 1, bottom left) was inactive (Dragoun et al. 2018).

With this in mind, we decided to also investigate the metal-free salalen ligand WQF 044 (Scheme 1, bottom middle). The catalytic activity and characterization of the compound is already known (Berkessel et al. 2007). Please note that the salalen WQF 044 is the direct salalen analogue of the salan THG 1213 investigated earlier (Dragoun et al. 2018). Additionally, no biological data representing the apoptotic activity of salalan ligands have been published so far. We were delighted to see that the salalen WQF 044 affected leukemia, lymphoma, mamma carcinoma and melanoma cells in a low micromolar range. We extended the number of cell lines to a human Ewing’s sarcoma cell line that we recently made resistant to vincristine. The main focus of interest was to find out the in-vitro cytotoxicity in resistant tumor cells and to compare the apoptotic activity of the compound with cytostatics including salans and salens that were published before. In fact, a proposal for the apoptotic mechanism of action of WQF 044 could be derived from our studies on a wide range of different tumor cells and modified cell lines.

## Results

### Proof of anti-proliferative effects of WQF 044 and cell death via apoptosis

**Fig. 1:**
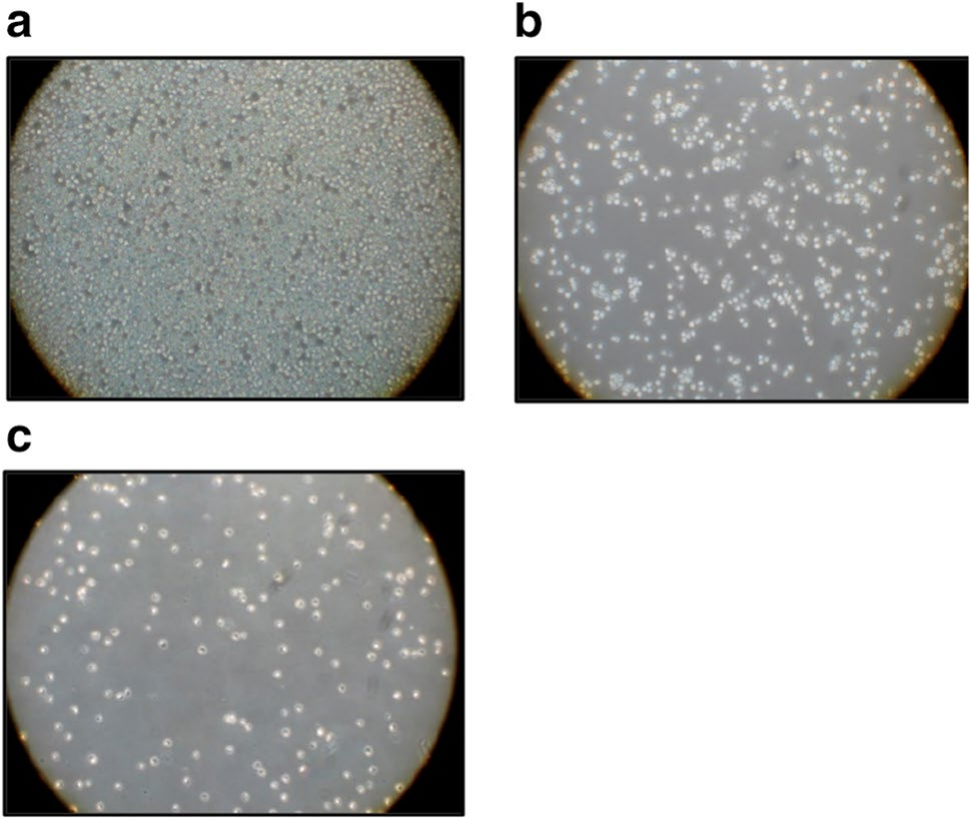
**a**–**c** Morphology of Nalm6 cells under the microscope after 72 h of incubation at 37 °C. **a** Control cells were left untreated. **b** Nalm6 cells were treated with 1 µM of WQF 044. **c** 10 µM of WQF 044 was pipetted on these cells

The salalen ligand WQF 044 inhibits proliferation and induces apoptosis in human B cell precursor leukemia cells (Nalm6). The following microscopic photos visualize the effect of WQF 044 on the Nalm6 cells after 72 h of incubation. In the control (Fig. 1a), a large number of Nalm6 cells can be observed. The cells are not apoptotic as they appear clear and round. Leukemia cells that were treated with 1 µM of WQF 044 are shown in Fig. 1b. Conspicuous is the lower number of cells. It is apparent that the cells look significantly damaged. They lost their circular and light appearance. Figure 1c clearly illustrates the high apoptotic effect on the cells after pipetting 10 µM of the compound. The few cells remaining are all damaged.

**Fig. 2:**
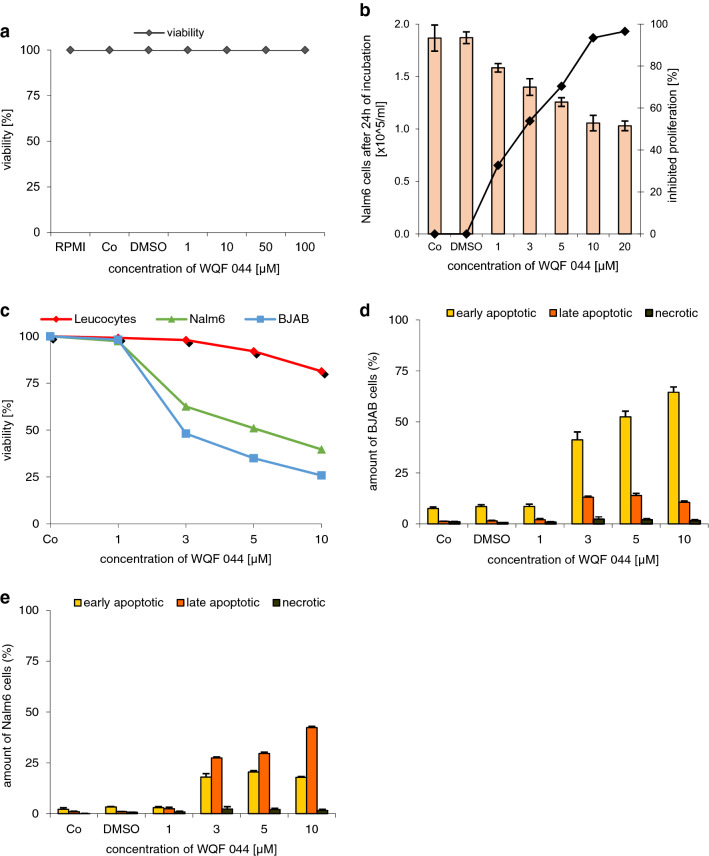
Inhibition of proliferation and excluding of necrosis. **a** Different concentrations of WQF 044 were pipetted on Nalm6 cells. After 3 h of incubation at 37 °C, an LDH release assay was made to determine the viability. Values are given as percentage of control (Co) (*n* = 3). **b** Nalm6 cells were treated with different concentrations of WQF 044 and incubated for 24 h. The CASY cell counter and analyzer system was used to measure the proliferation with three batches per concentration. The number of untreated control cells (Co) was set as 0% growth inhibition. Values are given as percentage of inhibition of cell proliferation (*n* = 3). **c–e** BJAB and Nalm6 cells and leucocytes were incubated with different concentrations of WQF 044 for 48 h. The viability, early and late apoptosis and necrosis were measured after Annexin V/PI staining by using flow cytometry. In the Annexin V/PI assay, vital cells (AnnV−/PI−) can be distinguished from early apoptotic (AnnV+/PI−), late apoptotic (AnnV+/PI+), and necrotic (AnnV−/PI+) cells. Three batches per concentration were used

The anti-proliferative potency of WQF 044 was proved by using the CASY cell counter. After pipetting 1–20 µM of the substance, an inhibited proliferation of over 95 percent was observed (Fig. 2b). IC_50_, the concentration of WQF 044 necessary to affect half maximal growth inhibition, is lower than 3 µM in Nalm6 cells. Furthermore, it is important that the cell death, triggered by WQF 044, is due to apoptosis and not necrosis. A lactate dehydrogenase (LDH) release measurement gives information about this fact. During necrosis, the cells lose their membrane integrity so that LDH can easily migrate out of the cells. This process does not happen during apoptosis within the first hours (Van Cruchten and Van Den Broeck 2002). We incubated WQF 044-treated Nalm6 cells for 3 h and made a LDH release assay that revealed no significant detection of LDH outside the cells (Fig. 2a) (Wieder et al. 1998). To specify the stage of apoptosis induced by WQF 044 an Annexin-V/propidium iodide (PI) assay was made. Annexin V binds to phosphatidylserine that is released in an early stage of apoptosis (Fadok et al. 2001; Schlegel and Williamson 2001). This exposure lasts until the final stage of apoptosis. PI is membrane impermeable. Therefore, cells that are devoid of PI staining show an intact membrane (van Engeland et al. 1998).

Next to the leukemic Nalm6 cells, Burkitt-like lymphoma cells (BJAB) were used for this experiment. First of all the data underlined that cell death induced by WQF 044 was due to apoptosis and not necrosis (Fig. 2d, e). After 48 h of incubation with WQF 044, it turned out that the highest amount of BJAB cells was in an early stage of apoptosis (Annexin V-positive, PI-negative) (Fig. 2d) whereas Nalm6 cells showed more late apoptosis after the same time (Annexin V/PI-double-positive), but still some early apoptotic cells (Fig. 2e). Furthermore, at a concentration of 10 µM, WQF 044 reduced the number of vital BJAB cells by approximately 75 percent and Nalm6 cells by 60 percent (Fig. 2c).

### WQF 044 overcomes multidrug resistance in leukemia cells

The overcoming of multidrug resistance (MDR) is an important quality of a new substance because MDR poses a huge problem in the treatment of relapsed malignant diseases (Uderzo et al. 2001). Various cellular-based mechanisms are responsible for the development of MDR in tumor cells. The excretion of P-glycoprotein (P-gp), encoded by the MDR1/ACBC1 gene, is one of these mechanisms (Gottesman et al. 2002; Krishna and Mayer 2000; Marques^ 2019^). It is an efflux pump in the cell membrane that transports drugs out of the cells resulting in a decrease of the cellular drug concentration (Gottesman and Pastan 1988; Lin and Yamazaki 2003; Mukhametov and Raevsky 2017). It has been proven that P-gp over-expression causes resistances to taxanes, anthracyclines and vinca-alkaloids because they are substrates of this transporter (Pieters et al. 1997; Wang et al. 2017). In our lab, we generated a vincristine resistant (NVCR) and a daunorubicin-resistant (NDau) Nalm6 cell line that are both characterized by an over-expression of P-gp (Rubbiani^ 2010^). They are additionally resistant to fludarabine, paclitaxel and colchicine (Kater et al. 2011; Kater 2012). Only recently, we found out that NDau cells are also resistant to cytarabine, mitoxantrone, idarubicin, doxorubicin, epirubicin, etoposide and the vinca alkaloids. Except of a cytarabine sensibility, NVCR cells have the same tested co-resistances as the NDau cells (Table 1).

**Table 1:** Co-Resistances of different tumor cell lines

	NVCR	NDau	BiBo	7CCA	RM82SiHoVCR
Cytarabine	S	R	R	R	R
Fludarabine	R	R	S	R	S
Cladribine	S	S	S	R	S
Clofarabine	S	S	S	R	S
Mitoxantrone	R	R	I	R	R
Idarubicin	R	R	S	R	S
Daunorubicin	R	**R**	R	R	R
Doxorubicin	R	R	S	**R**	R
Epirubicin	R	R	I	R	R
Etoposide	R	R	S	R	I
4-OH-Cyclophosphamide	S	S	S	R	S
Methotrexate	S	S	S	S	S
Vindesine	R	R	R	R	R
Vinorelbine	R	R	R	R	R
Paclitaxel	R	R	R	R	R
Vinblastine	R	R	R	R	R
Vincristine	**R**	R	**R**	R	**R**
Fluoruracil	S	S	S	S	I
Resistant mechanisms	P-gp over-expression	P-gp over-expression	Bcl-2 over-expression	Caspase-3 under-expression	CD95 under-expression, caspase-8 downregulation

DNA-fragmentation was measured as described with the wild-type cell lines and its resistant cell line. The written cytostatics were pipetted on the cells in different concentrations (*n* = 3). Apoptotic effects were compared. Cells were resistant (R) if the apoptotic effect in the resistant cell line was half times lower than in the main cell line in a dose-dependent manner. Intermediate (I) stadium was chosen if apoptosis was significantly lower in the resistant cell line, but still higher than the half-value of the non-resistant cell line. Otherwise the cells were sensible (S) to the cytostatic. The primary resistant mechanisms of the cell lines are written with an R in bold

**Fig. 3:**
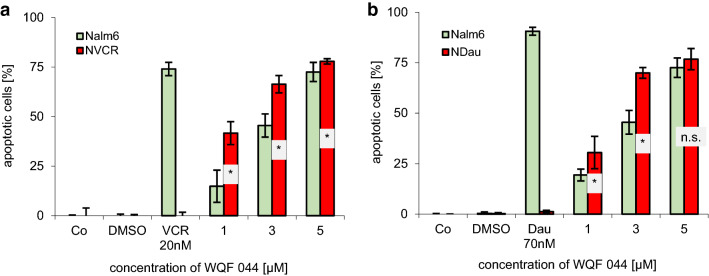
Overcoming of MDR is proved by treating resistant cells and control Nalm6 cells with different concentrations of WQF 044. Untreated cells were left as control (Co). Incubation time was 72 h at 37 °C. DNA fragmentation was measured by flow cytometric analysis. Values are given in percent ± SD (*n* = 3). **a** DNA fragmentation of Nalm6 cells was compared with vincristine-resistant Nalm6 cells (NVCR). To prove the resistance, both cell lines were treated with vincristine (VCR). **b** The experiment was repeated with Nalm6 cells and daunorubicin-resistant NDau cells and resistance is shown by comparing the apoptotic effects in daunorubicin (Dau) treated cells. Comparison of data was calculated by a one-tail *t* test: *p < 0.05; *ns* not significant

DNA fragmentations effected by WQF 044 in the resistant cell lines compared to the Nalm6 cell line revealed that this ligand has the potential to overcome the MDR. The apoptotic effects were even significantly higher in the resistant cell lines than in the control Nalm6 cells after exposing them to 1 to 5 µM of WQF 044 (Fig. 3a, b).

### Bcl-2 over-expression in Burkitt-like lymphoma and human melanoma cells reduces activity of WQF 044

**Fig. 4:**
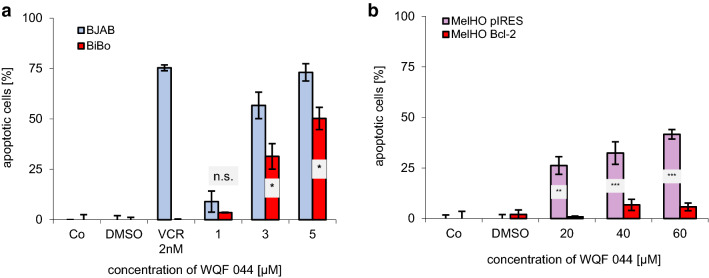
Dependence of a Bcl-2 over-expression in vincristine-resistant BiBo cells and pIRES-Bcl-2-vector transfected MelHO cells. After 72 h of incubating the cells at 37 °C, DNA fragmentation was measured by flow cytometric analysis. Control cells were left untreated (Co). Apoptotic cells were displayed as percentage of the means of three separate samples ± SD. Comparison of data was calculated by a one-tail t-test. Results have been asterisked as follows: *p < 0.05; **p < 0.01; ***p < 0.001; *ns* not significant. **a** BJAB and BiBo cells were treated with different concentrations of WQF 044. Additionally, both cell lines were treated with vincristine (VCR) to prove the resistance. **b** WQF 044 was pipetted on MelHO pIRES und MelHO Bcl-2 cells in three different concentrations. Higher concentrations than in Nalm6 and BJAB cells were used due to the lower sensitivity of these adherent cells

Another mechanism of MDR is the over-expression of the anti-apoptotic protein Bcl-2 in tumor cells (Krishna and Mayer 2000). At this point, BJAB cells and its vincristine-resistant cells (BiBo) played a main role because the BiBo cells are characterized by an Bcl2-over-expression (Kater et al. 2011). The cells further have co-resistances to cytarabine, daunorubicin, vindesine, vinorelbine, vinblastine and paclitaxel (Table 1). A DNA fragmentation with WQF 044 in these two cell lines gave further information about the working mechanism of the substances. First of all, it can be said that WQF 044 induces apoptosis in BJAB cells in a dose-dependent manner (Fig. 4a). In comparison to that the apoptotic effects in BiBo cells were significantly lower than in the BJAB cells, but at least values up to 50 percent at 5 µM of WQF 044 could be reached. The same phenomenon was observed in the analysis of a DNA fragmentation with special human melanoma cells (MelHO). The MelHO pIRES cell line was stably transfected with the pIRES vector. Compared to this the MelHO Bcl-2 cells had the pIRES-Bcl-2-vector included. They strongly over-express the anti-apoptotic Bcl-2 protein (Jesse et al. 2009). Figure 4b clearly demonstrates that WQF 044 had no effect on the MelHO Bcl-2 cell line, but induced apoptosis in the MelHO pIRES cells with a high significance compared to the MelHO Bcl-2 cells, respectively.

### WQF 044 regulates mitochondria-related Bcl-2 family proteins, DIABLO and cytochrome c

Even though a dependence of WQF 044 on the Bcl-2 over-expression can be observed, we found several gene expressions, including members of the Bcl-2 family, in a polymerase chain reaction (PCR) analysis of WQF 044 treated Nalm6 cells. The analysis revealed a downregulation of the anti-apoptotic Bcl-2 by 18-fold and a 5-times upregulation of the Bcl-2-associated X protein (BAX) that is pro-apoptotic (Table 2) (Edlich 2018; Garner et al. 2017). Thus, we assume that WQF 044 has an influence on Bcl-2 even though higher concentrations are needed to reach apoptotic effects.

**Table 2:** PCR analysis of WQF 044 treated Nalm6 cells

Genes over-/under-expressed	Fold-change	Fold-regulation
Bcl-2	0.0545	− 18.3537
BAX	4.6332	4.6332
CD95	0.0236	− 42.459
Cytochrome C	42.5769	42.5769
DIABLO	15.9115	15.9115
FADD	0.1758	− 5.6883

WQF 044 treated Nalm6 cells were incubated for 16 h at 37 °C. For PCR analysis the apoptosis-specific RT2 profiler PCR expression array (SuperArray PAHS-012Z; SABiosciences Corporation, Frederick, MD, USA) was used. Fold-Change (2^(−ΔΔCt)) is the normalized gene expression (2^(−ΔCt)) in the Test Sample (WQF 044 treated Nalm6 cells) divided the normalized gene expression (2^(−ΔCt)) in the Control Sample (untreated Nalm6 cells). Fold-Regulation represents fold-change results in a biologically meaningful way. Fold-change values greater than one indicate a positive- or an up-regulation, and the fold-regulation is equal to the fold-change. Fold-change values less than one indicate a negative or down-regulation, and the fold-regulation is the negative inverse of the fold-change

The PCR array analysis also produced other gene expressions that lead to the pathway in which WQF 044 intervened. An over-expression of the IAP-binding mitochondrial protein (DIABLO) and cyctochrom c, somatic (CYCS) was observed (Table 2). These proteins, including Bcl-2 family members, are part of the intrinsic apoptotic pathway that is mitochondria-dependent (Won^ 2018^). However, apoptosis can also be triggered by the death receptor-dependent extrinsic pathway (Nair et al. 2014).

### The mitochondrial membrane potential is reduced by WQF 044

**Fig. 5:**
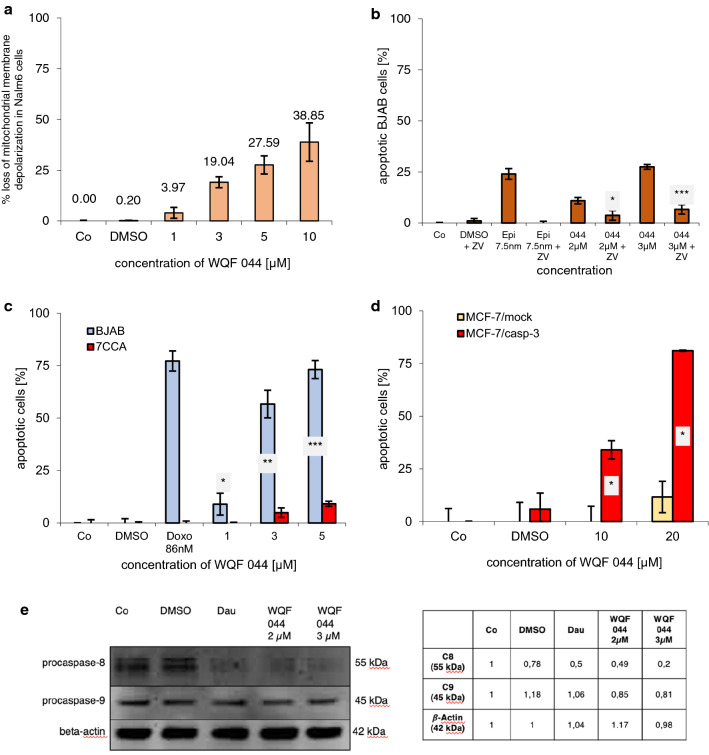
Caspase dependence of WQF 044. Control cells (Co) were left untreated or treated with DMSO. Comparison of data was calculated by a one-tail *t* test. Results have been asterisked as followed: *p < 0.05; **p < 0.01; ***p < 0.001. **a** After incubating Nalm6 cells for 48 h hours with different concentrations of WQF 044, the mitochondrial permeability transition was measured by flow cytometric analysis. Values are given as percentage of cells with low ΔΨ_m_ ± SD (*n* = 3). **b** BJAB cells were treated with 2 µM and 3 µM of WQF 044, alone and in combination with the pancaspase-inhibitor zVAD-fmk (ZV). As control 7.5 nM of epirubicin (Epi) were pipetted alone and in combination with ZVAD-fmk. After 72 h of incubation at 37 °C, DNA fragmentation was measured by flow cytometric analysis. Values were given in percentage of apoptotic cells ± SD (*n* = 3). Statistical comparison of data was between the single WQF 044 group and the combined treatment with ZV, respectively. **c** BJAB and 7CCA cells were incubated with different concentrations of WQF 044 for 72 h. DNA fragmentation was measured by flow cytometric analysis. Apoptotic cells were displayed as percentage of the means of three separate samples ± SD. Doxorubicin (Doxo) was applied at 86 nM to show the resistance. **d** MCF-7/mock cells were compared with MCF-7/casp-3 cells in their induction of apoptosis after incubating them with 10 and 20 µM of WQF 044 for 72 h (*n* = 3). As in the MelHO cells, higher concentrations than in Nalm6 and BJAB cells were used due to the lower sensitivity of these adherent cells. **e** WQF 044 induces caspase-8 (C8) and caspase-9 (C9) activation in Nalm6 cells. Daunorubicin (Dau) was used as positive control. Nalm6 cells were treated with 2 µM and 3 µM of WQF 044. All cells were incubated for 48 h at 37 °C. The separation of 20 µg cytosolic proteins was done by SDS-PAGE, followed by subjecting them to the western blot analysis. Immunblotting was then done with an anti-C8 and anti-C9 antibody. 43 kDa β-actin was detected to prove equal loading. The western blot quantification was calculated using GeneTools (Syngene). The control was set as 1. DMSO, Dau and WQF 044 2 and 3 µM were compared with the control, respectively

The activation of the intrinsic pathway results in a loss of the mitochondrial membrane potential (Lambert et al. 1989). To investigate the influence of WQF 044 on the mitochondrial permeability transition that can already be seen in the results of the PCR array analysis, Nalm6 cells were stained with a JC-1 dye. Flow cytometric determination showed a decreased fluorescence of Nalm6 cells, reflecting mitochondrial permeability. Figure 5a clearly illustrates the loss of mitochondrial membrane depolarization, beginning from 3 µM of WQF 044.

### Caspase-dependence of WQF 044

The protease caspase-3 is a key component of the apoptotic pathway. During apoptosis, it is taking part in the proteolytic cleavage of many key proteins (Cohen 1997). To investigate whether WQF 044 develops its apoptotic effects via an activation of caspase-3, we tested the substance on special cell lines. One cell line is the doxorubicin-resistant BJAB cell line (7CCA) that is characterized by a caspase-3 under-expression (Dragoun et al. 2018). Multiple co-resistances of 7CCA were found to cytarabine, fludarabine, cladribine, clofarabine, mitoxantrone, idarubicin, daunorubicin, epirubicin, etoposide, 4-OH-cyclophosphamide, vindesine, vinorelbine, paclitaxel, vinblastine and vincristine (Table 1). Figure 5c clearly illustrates that WQF 044 could not affect the 7CCA cells, whereas the regular BJAB cells were highly apoptotic at WQF 044 concentrations of 3 to 5 µM.

To further support that WQF 044 is dependent of caspase-3, we used the human breast adenocarcinoma cell line MCF-7/mock and its modified cells called MCF-7/casp-3. It is known that MCF-7/mock cells lack the enzyme caspase-3 (Engels et al. 2005). The second one has caspase-3 later incorporated in the cells so that they stably express this protease. In these MCF-7/casp-3 cells, the DNA fragmentation grade increased from 34 percent at 10 µM of WQF 044 to 81 percent at 20 µM (Fig. 5d). In comparison, exposing MCF-7/mock cells to WQF 044 resulted in no induction of apoptosis. At 20 µM of WQF 044, less than 12 percent of the cells were apoptotic.

Our investigations of WQF 044 in caspase-3 over- and under-expressed cell lines were supported by using the pancaspase-inhibitor zVAD-fmk. The treatment of BJAB cells with WQF 044 and zVAD-fmk, alone and in combination, revealed an inactivation of the salalen ligand. WQF 044 could not induce apoptosis under the influence of the inhibitor in a dose-dependent manner (Fig. 5b). The apoptotic effects were significantly lower in the combination treatment with the inhibitor and 2 µM of WQF 044, and even very significantly reduced further when using 3 µM of WQF 044 with zVAD-fmk. This result underlines the dependence of WQF 044 on the broad caspase-induced cell-death.

A western blot analysis, after treating Nalm6 cells with 2 and 3 µM of WQF 044 for 48 h, revealed smaller procaspase-9 (49 kDa) and procaspase-8 (55 kDa) bands in the exposed cells compared to the control cells (Fig. 5e). The western blot quantification illustrated the lower number of proteins of inactivated caspase-8 and caspase-9 in comparison to the control cells. The processing of caspase-9 underlined the activation of the mitochondrial pathway by WQF 044.

### WQF 044-induced apoptosis is independent of the extrinsic pathway

**Fig. 6:**
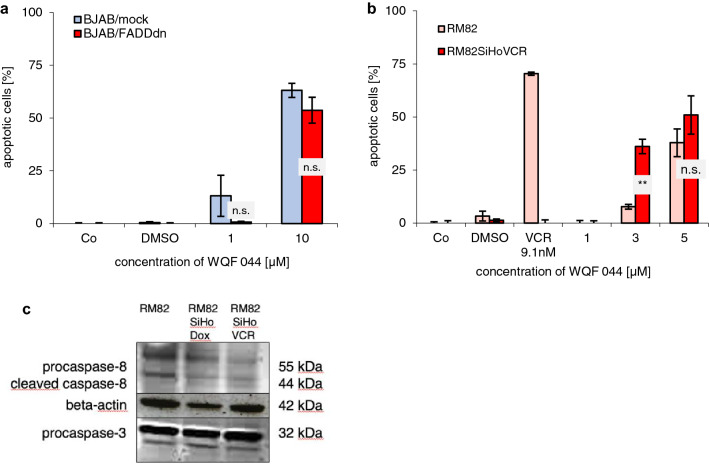
Exclusion of the extrinsic apoptotic pathway. Incubation time for DNA fragmentation was 72 h at 37 °C. It was measured by flow cytometric analysis. Values were given in percentage of apoptotic cells ± SD (*n* = 3). Control cells (Co) were left untreated. **a** BJAB mock and BJAB/FADDdn cells were exposed with 1 and 10 µM of WQF 044. The differences between the values of WQF 044-treated BJAB/mock and BJAB/FADDdn cells in the two concentrations were not significant (n.s.) with *p* > 0.05. **b** RM82 and vincristine (VCR)-resistant RM82SiHoVCR cells were treated with different concentrations of WQF 044. Comparison of data was performed using a one-tail *t* test. Results have been asterisked as **p < 0.01; *ns* not significant. **c** Presence of caspase-8 and -3 in untreated Ewing’s sarcoma cells. For this paper, we focus on the RM82 and RM82SiHoVCR cell line. The separation of 20 µg cytosolic proteins was done by SDS-PAGE, followed by subjecting them to western blot analysis. Immunoblotting was then done with anti-caspase-3 and anti-caspase-8 antibodies. 42 kDa β-actin was detected to prove equal loading

Significant involvement of the extrinsic pathway, characterized by an activation of caspase-8, was excluded in different ways. BJAB/FADDdn cells were transfected with pcDNA3-FADD−/−. They are expressing a dominant negative FADD mutant that is lacking the death domain. The BJAB/mock cells have a pcDNA3-Primer without the FADDdn gene. The values of WQF 044-treated apoptotic BJAB/FADDdn cells were not significantly higher than these in BJAB/mock cells (Fig. 6a).

Superficially, an activation of caspase-8 in the western blot analysis was inconsistent with the results of the BJAB/FADDdn cells. However, a previous study demonstrated that caspase-8 can be activated by caspase-3 over the intrinsic pathway; hence its activation is independent of the CD95 death-inducing signaling complex (Wieder^ 2001^).

These results were further supported by a DNA-fragmentation with a Ewing’s sarcoma cell line from a primary tumor in the femur, called RM82, that we newly made resistant to vincristine (RM82SiHoVCR) (Ottaviano 2010; van Valen et al. 1992). Polymerase chain reactions and western blot analysis revealed a sixfold CD95-underexpression (data not shown) and a downregulation of procaspase-8 (Fig. 6c) in the vincristine-resistant RM82 cells, whereas caspase-3 bands did not distinguish between the primary tumor and the resistant cell line. Next to their vincristine-resistance, RM82SiHoVCR cells are resistant to several vinca alkaloids, anthracyclines (Table 1) and platins. CD95, also called Fas, is a pro-apoptotic member of the extrinsic pathway (Lavrik and Krammer 2012; Schmidt et al. 2015; Strasser et al. 2009). Thus, apoptosis of RM82SiHoVCR seems to be blocked over the extrinsic pathway. Van Valen et al. earlier described that wild-type RM82 cells can be affected by tumor necrosis factor-related apoptosis-inducing ligand (TRAIL), a member of the extrinsic pathway of apoptosis (Van Valen et al. 2000). Figure 4b clearly demonstrates that the induction of apoptosis by WQF 044 was not disrupted by the CD95-under-expression in vincristine-resistant Ewing’s sarcoma cells. At a WQF 044 concentration of 3 µM, the value of apoptotic RM82SiHoVCR cells was even significantly higher than in regular RM82 cells.

### WQF 044 sensitizes BJAB cells to vincristine

**Fig. 7:**
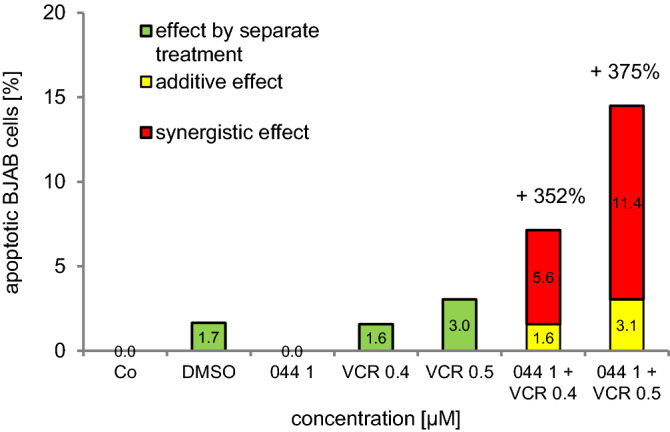
Synergistic effects of WQF 044 (044) and vincristine (VCR). BJAB cells were treated with 1 µM of WQF 044 and 0.4/0.5 µM of vincristine, alone and in combination. Control cells (Co) were left untreated or with DMSO. After 72 h of incubation at 37 °C, induction of apoptosis was measured by flow cytometric analysis of nuclear DNA fragmentation. Values were given in percentage of apoptotic cells and were expressed at means ± SD (*n* = 3). To determine synergistic effects, the fractional product was calculated

The use of polychemotherapy results in lower side effects of the selected cytostatic drugs during cancer treatment. Therefore, the compound WQF 044 was tested in combination with vincristine to see if it had the potential to sensitize BJAB cells. Vincristine is a main chemotherapeutic in the fight against Burkitt lymphoma (Dunleavy 2013; Painschab 2019). Thus, the cells were exposed separately to WQF 044 and vincristine and to a combination of both cytostatic agents. Very low concentrations were used that did not affect the cells in single use. The effects are shown in Fig. 7. Synergistic effects of 352 percent after combining 1 µM of WQF 044 with 0.4 µM of vincristine were observed. An even stronger synergism appeared after pipetting 1 µM of WQF 044 plus 0.5 µM of vincristine on BJAB cells (375 percent).

### WQF 044 is highly selective to tumor cells

**Fig. 8:**
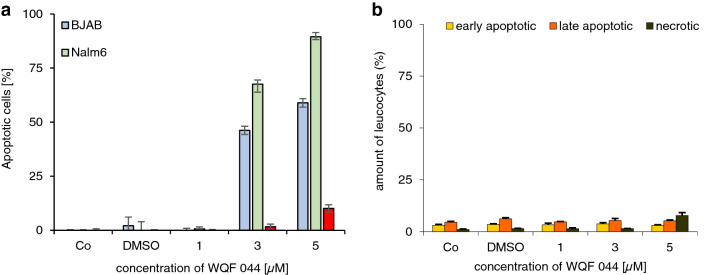
Selectivity of WQF 044 to cancer cells. **a** A DNA fragmentation of treated BJAB, Nalm6 and non-malignant leucocytes was done. Cells were treated with 1 to 5 µM of WQF 044 and incubated for 72 h at 37 °C. The number of apoptotic cells was measured by flow cytometric analysis using three batches per concentration. Values were given in percentage and were expressed at means ± SD. **b** After incubation at 37 °C for 48 h with 1 to 5 µM of WQF 044, early and late apoptosis and necrosis were measured in leucocytes after Annexin V/PI staining by using flow cytometry (*n* = 3). AnnV+/PI– cells were defined as early apoptotic, AnnV+/PI+ as late apoptotic and AnnV–/PI+ as necrotic cells

Remarkably, we found out that WQF 044 had nearly no apoptotic effect in healthy human leucocytes. We compared a DNA fragmentation of the substance in leucocytes with those in BJAB and Nalm6 cells. While the cancer cells were highly affected by WQF 044 in a dose-dependent manner, leucocytes showed no significant levels of apoptosis in WQF 044 concentrations from 1 to 5 µM (Fig. 8a). As it turned out in the measurement of early/late apoptosis and necrosis, WQF 044 did not affect the leucocytes in a dose-dependent manner (Fig. 8b). At a WQF 044 concentration of 5 µM, less than 10 percent of the cells were either apoptotic or necrotic whereas the viability of the cells was at 92 percent (Figs. 2c, 8b).

## Discussion

The discovery of novel organic compounds is of high interest since the discovery of the high cytostatic metal complex cisplatin. Severe side effects such as nephrotoxicity, allergic reactions and gastrointestinal symptoms often limit chemotherapy with cisplatin (Dasari and Tchounwou 2014). Therefore, the metal-free salalen ligand WQF 044 might be a potential alternative with less toxicity.

Especially after long-term chemotherapy, MDR represents a huge problem that causes therapy failure (Baguley 2010). In clinical use, cytostatic agents that modulate ABC transporters have not always proven as efficient as they seemed during their laboratory investigations (Li 2017). Thus, finding new substances with the potential to overcome MDR is a very important goal in oncology research. The examination of the metal-free ligand WQF 044 revealed very promising characteristics of the substance as a future cytostatic drug. An impressive overcoming of MDR in daunorubicin- and vincristine-resistant Nalm6 cells was observed (Fig. 3a, b). The substance defeated the over-expression of P-gp. Moreover, quite remarkable anti-proliferative effects of WQF 044 in leukemia cells were detected in a low micromolar range (Fig. 2b).

The synergetic effects of WQF 044 and vincristine in BJAB cells make the compound even more attractive as a substance that overcomes the defense barrier of the acute lymphoblastic leukemia and Burkitt-lymphoma that cannot be successfully treated with typical common therapies (Fig. 7).

Potential anti-cancer substances often fail in clinical use due to high toxicity. For example, colchicine was used in low concentrations to treat gout and familial Mediterranean fever, but the higher concentrations, that are necessary for anti-cancer treatment, resulted in too many side effects (Kumar et al. 2017). Furthermore, several metal complexes were very promising for anti-cancer treatment, but their *in-vivo* toxicity often limited their use (Liang et al. 2017). In contrast, the substance WQF 044 is a metal-free ligand, thus avoiding metal-induced toxicity. As a most exciting consequence, no apoptotic effects were seen upon treating the healthy human leucocytes with WQF 044, compared to the high apoptotic values in leukemia and lymphoma cells (Figs. 2c, 8a, b). In comparison, leukemia and lymphoma cells lost their viability and showed early and late apoptosis after treating them with WQF 044 (Fig. 2c–e).

Other metal ligands have already been tested in tumor cells. As mentioned in the introduction, the metal-free salan-type compound THG 1213 has been examined and showed anti-proliferative and apoptotic effects in Nalm6 and solid tumor cells and their resistant cell lines in low micromolar concentrations. In contrast, the salen ligand THG 1212 did not affect tumor cells (Dragoun et al. 2018).

Next to the experiments that were done with THG 1213, we widened the examination of WQF 044 with several experiments. Due to our high interest in new metal complexes and their ligands, we focused on cells of a Ewing’s sarcoma. The well-known platin derivate cisplatin is used in relapsed treatment of Ewing’s sarcoma patients, but strong side effects limit their use (van Maldegem 2015). Therefore, the impressive apoptotic effects of WQF 044, even in resistant Ewing’s sarcoma cells, are an important finding (Fig. 6b). The substance may be an alternative treatment in relapsed Ewing’s sarcoma that still has a very frustrating survival-prognosis (Cotterill et al. 2000).

We further observed that the working mechanisms of WQF 044 and THG 1213 are different: THG 1213 could not affect the BJAB/FADDdn cells in the same way than regular BJAB cells, underlining their dependence of the extrinsic pathway, whereas WQF 044 showed a clear independence of the extrinsic pathway of apoptosis as it was not dependent of CD95 (Fig. 6a) (Dragoun et al. 2018). CD95 (APO-1/Fas) is a death receptor and part of the death-including complex that activates caspase-8 over the adapter molecule FADD (Krammer 2000). The independence of the WQF 044-induced apoptosis on the extrinsic pathway was supported by the results of the PCR array analysis: a sixfold under-expression of FADD and a 42-fold under-expression of CD95 were observed what is a clear point against the main involvement of the extrinsic pathway (Table 2).

Especially the fact that the salan THG 1213 induced apoptosis in a low micromolar range, but the salen THG 1212 did not affect the cells made the exploration of the corresponding salalen WQF 044 most interesting. The different results of THG 1213 and WQF 044 underline that even small differences in the chemical structure of a compound can cause completely different results in human cells. Earlier studies on other salans showed that these compounds developed anti-proliferative effects in MCF-7 and other human breast cancer cells at concentrations of 1–2 µM (Gao et al. 2007), whereas WQF 044 needed higher concentrations to affect MCF-7 cells. THG 1213 could not affect MCF-7 cells at all in this micromolar range (Dragoun et al. 2018). Future in-vivo experiments are intended to probe whether the metal-free ligands WQF 044 and THG 1213 show different efficiency on this level.

The examination of other substances illustrates that it cannot be predicted a priori whether a ligand or a metal complex cause better anti-cancer effects. For example, Kilic et al. (2018) found that different hemi-salen ligands showed higher anticancer activity in four different cancer cell lines, whereas related triboron complexes did not affect the cells in the same way (Kilic et al. 2018). Interestingly, Ghanbari (2014) observed that the increase in aromatic rings on the bridge between d-amino groups caused more apoptotic activity of their Fe(III)-salen-like complexes (Ghanbari et al. 2014). Once again, both the ligand structure and the position of the substituent of the salen ligand can modify the effects on cancer cells.

In the case of the molybdenum complexes described by Dragoun et al. (2018), the complexes did not affect the tumor cells in the same way the ligands THG 1213 and WQF 044 did (Dragoun et al. 2018). In comparison to that, another study illustrated that a cobalt(III) salen complex had cytotoxic effects in BJAB and Nalm6 cells, but the corresponding ligand could not induce apoptosis in the tumor cells (Hopff et al. 2020). Furthermore, ruthenium(II) complexes had cytotoxicity effects in MCF-7 and human prostate tumor cells (DU-145) in a low micromolar range, whereas their metal-free-ligand did not provoke the same outcome (Correa^ 2016^).

Due to its high anti-cancer potential, we were very interested in the mode of action of WQF 044 and found out that the intrinsic pathway played a main role. The mitochondrial membrane potential was reduced in WQF 044 influenced Nalm6 cells (Fig. 5a). The high permeability of the mitochondria membrane results in a release of cytochrome c into the cytosol (Kroemer and Reed 2000). This activates procaspase-9, among other proteins. The downstream caspases, especially caspase-3 and caspase-8, are stimulated, so that they lead to DNA fragmentation and apoptosis (Hengartner 2000; Herr and Debatin 2001; Nagata 2000). The consumption of procaspase-8 and procaspase-9 after treating Nalm6 cells with WQF 044 was detected in the western blot analysis (Fig. 5e). The fact that caspase-8 was activated by WQF 044, but the substance did not mainly act over the extrinsic pathway was explained by the phenomenon of a caspase-3 induced caspase-8 activation as described (Wieder et al. 2001).

Furthermore, besides the blocking of P-gp, the compound acted caspase dependent. The extremely low apoptotic effects in caspase-3 under-expressed 7CCA and caspase-3 defective MCF-7/mock cells clearly illustrated that the protease is necessary for WQF 044 to develop its effects on the cells (Fig. 5c, d).

The strong inhibition of WQF 044-induced apoptotic effects by the pancaspase-inhibitor zVAD-fmk clearly supported the caspase-dependent apoptotic mechanism of the compound (Fig. 5b). The zVAD-fmk inhibitor did not completely block apoptosis in WQF 044 treated BJAB cells, whereas the positive control with epirubicin showed no apoptosis. This result points to the involvement of other members of the apoptotic pathway in the mechanism of action of the compound WQF 044.

A 43-fold over-expression of cytochrome c was detected in the PCR array analysis (Table 2). WQF 044 also over-expressed the direct IAP (inhibitor of apoptosis)-binding protein (DIABLO) by 15-fold (Table 2). DIABLO is part of the intrinsic pathway as it is released from the mitochondria. It neutralizes the anti-apoptotic activity of IAP family members (Herr and Debatin 2001). The release of pro-apoptotic components like cytochrome c and DIABLO from the mitochondria is regulated by members of the Bcl-2 family. They influence apoptosis by changing the mitochondria membrane permeability (Antonsson and Martinou 2000; Liu and Hengartner 1999). We found that WQF 044 is dependent on the anti-apoptotic Bcl-2. The over-expression of Bcl-2 in BiBo and MelHO-Bcl2 cells reduced the apoptotic effects of WQF 044, underlining the involvement of the intrinsic pathway of apoptosis (Fig. 4a, b). Even though WQF 044 did not have the same apoptotic effects in BiBo cells compared to BJAB cells, it still induced apoptosis and overcame the Bcl-2 over-expression (Fig. 4a). This can be explained by the fact that WQF 044 inhibited Bcl-2, as the compound evoked an 18-fold under-expression of this anti-apoptotic protein (Table 2). However, WQF 044 did not break the over-expression of Bcl-2 by 30-fold, existing in the MelHO/Bcl-2 cells, in a dose-dependent manner (Fig. 4b) (Onambele 2010). Another interesting observation was the fivefold over-expression of BAX in the PCR array analysis (Table 2). This is in line with the inhibition of the WQF 044-induced apoptotic effects when anti-apoptotic Bcl-2 is predominant because then it inhibits BAX. Once activated, BAX directly supports the mitochondrial outer membrane permeabilization resulting in a release of killing effectors into the cytosol (Renault et al. 2013).

With our investigations of the salalen ligand WQF 044, we clearly detected the apoptotic mechanisms in different tumor cells and pointed out a main involvement of the intrinsic pathway of apoptosis. Next to the control of the intrinsic pathway of apoptosis by releasing proapoptotic factors, mitochondria own other functions to influence cell death (Orrenius et al. 2003). Therefore, the exact way of how a substance activates the mitochondrial mechanisms of apoptosis can differ. The metal ion reservoir can be depleted or the production of reactive oxygen species (ROS) can be increased above a cytotoxic threshold (Bao 2019; Nam et al. 2018; Yang et al. 2016). Further investigations in this direction are beyond the focus of this manuscript. It will surely to address these issues in future experiments.

## Conclusion

Overall, our investigations revealed that salalen ligands, exemplified by compound WQF 044, are quite promising for anti-cancer treatment. Especially the low cytotoxic effects in healthy human leucocytes and the wide range of tumors, that WQF 044 can combat, make the substance highly interesting for future use as a chemotherapeutic. Additionally, we gained new insights into the in-vitro activity of this compound by underlining the mitochondrial function in cancer cells, resulting in a new approach to fight against resistant cancers, especially in childhood.

## Methods

### Materials

Rnase A was from Qiagen (Hilden, Germany). Propidium iodide (50 µg/ml) was from Serva (Heidelberg, Germany). Further the zVAD-fmk (pancaspase/ panprotease) inhibitor was used. All common cytostatics were provided by the Children’s Hospital of the City Cologne, Amsterdamer Straße. The cytostatic agents were freshly dissolved as stock solutions in DMSO prior to the experiments. They were diluted with the respective cell culture media or buffer during the assay procedures. The substance WQF 044 was dissolved in a 40-mM stock solution of DMSO. Next to the regular control cells in the experiments, some cells were incubated with an equal amount of DMSO only, as DMSO control. The results showed similar effects to those obtained in the untreated controls.

WQF 044 was synthesized and characterized according to Berkessel et al. (2007).

### Cell lines and cell cultures

We used the human b cell precursor leukemia cell line Nalm6 (AG Henze, Charité, Berlin), the Burkitt-like lymphoma BJAB/mock cells (Prof. Dr. S. Fulda, University of Ulm), the BJAB/FADDdn cells (Peter Daniel, Charité Berlin), the breast adenocarcinoma MCF-7/mock and MCF-7/casp-3 cells (Prof. Dr. R. Jänicke, University of Düsseldorf) and the human melanoma MelHO pIRES and MelHO Bcl-2 cells (Dr. Eberle, Charité, Berlin). Nalm6 and BJAB cells were made resistant in our lab by exposing them to increasing concentrations of the current cytostatic drug. RM82 is a human Ewing’s sarcoma cell line from F. Van Valen, University of Muenster, Germany that was exposed to increasing concentrations of vincristine to make the cells resistant (RM82SiHoVCR).

The cell lines were incubated in 250 ml cell culture bottles at 37 °C. RPMI 1640 medium (GIBCO, Invitrogen, Karlsruhe, Germany) was used for the suspension cells. Heat inactivated fetal calf serum (FCS, 10%, v/v), L-glutamine (0.56 g/l), penicillin (100,000 i.u.) and streptomycin (0.1 g/l) were added. The adherent cells were grown in DMEM supplemented with FCS (10%, v/v) and geniticine (0.4 mg/ml). We took care of the cell cultures twice a week and diluted them to a concentration of 1 × 10^5^ cells/ml. 24 h before using the cells for experiments, they were adjusted to 3 × 10^5^ cells/ml to have standard conditions in growing. Immediately before the start of experiments, cells were diluted to 1 × 10^5^ cells/ml.

### Measuring the inhibition of cell proliferation by CASY cell counter and analyzer system

CASY cell counter and analyzer system from Roche were used to determine cell count and viability with specifically defined settings for the current cell lines. The system measures the cell concentration in three different size ranges that include cell debris, dead cells and viable cells (Voisard et al. 1991). Before measuring, all cells were treated with different concentrations of WQF 044. Some cells were left untreated or with DMSO as control. Incubation time was 24 h at 37 °C. Then, cells were resuspended and 100 μl of each well was diluted in 10 ml of CASYton (ready-to-use isotonic saline solution) for an immediate automated count of the cells. We defined the control group of the cells as 100% growth meaning that a cell concentration not higher than at the beginning of the experiment reveals maximum inhibition of cell proliferation.

### LDH release assay for excluding necrosis

To make sure that cell death was due to apoptosis and not necrosis, a measurement of the lactate dehydrogenase (LDH) release was made. Nalm6 cells were exposed to different concentrations of WQF 044 and incubated for 3 h. With the help of the Cytotoxicity Detection Kit from Roche (Mannheim, Germany), the release of LDH out of the cells was measured in the cell culture supernatants. After centrifugation at 350*g* for 5 min, 20 µl of cell-free supernatants was diluted with 80 µl phosphate-buffered saline (PBS) supplemented with 100 µl reaction mixture containing 2-(4-iodophenyl)-3-(4-nitrophenyl)-5-phenyl-tetrazolium chloride (INT), sodium lactate, NAD^+^ and diaphorase. Time-dependent formation of the reaction product was quantified photometrically at 490 nm. The maximum amount of LDH release was determined after lysis of the cells using 0.1 percent Triton X-100 in culture medium and set to represent 100 percent cell death.

### Annexin V/propidium iodide binding assay

The Annexin-V-Fluos Staining kit (Roche, Mannheim, Germany) was used to detect early and late apoptosis and necrosis. Nalm6 and BJAB cells and healthy human leucocytes were incubated with different concentrations of WQF 044 for 48 h at 37 °C. The preparation of the samples was made according to the instructions of the manufacturer. The data were analyzed by using the FACScan (Becton Dickinson, Heidelberg, Germany), equipped with the CELL Quest software. Early apoptosis was defined as Annexin V-positive and PI-negative and late apoptosis as Annexin V/PI-double-positive. Vital cells were Annexin V/PI-double-negative (Brauchle et al. 2014; Wlodkowic et al. 2011). Annexin V-negative and PI-positive marked necrotic cells that were damaged during the isolation procedure (van Engeland et al. 1998).

### Measurement of DNA fragmentation

For detecting the percentage of apoptotic cells, different concentrations of WQF 044 were pipetted on the cells before incubating them for 72 h at 37 °C. Then, adherent cells were washed with 180 µl Phosphate-buffered saline (PBS) and treated with trypsin for 5 min at 37 °C. A centrifugation at 1500 rpm helped to collect all cells. Then, they were fixed in 200 µl PBS/2% (v/v) formaldehyde on ice for 30 min, continued by a centrifugation at 1500 rpm for 5 min by 4 °C. After incubating them with 180 µl ethanol/PBS (2:1, v/v) for 15 min, followed by a 5-min centrifugation at 1500 rpm, cells were resuspended in 50 µl PBS containing 40 μg/ml Rnase A that for 30 min at 37 °C. Cells were centrifuged at 1500 rpm for 5 min for a last time; then they were finally resuspended in 200 µl PBS containing 50 μg/ml propidium iodide. As described, the nuclear DNA fragmentation on the single cell level was measured by a modified cell cycle analysis (Essmann et al. 2000). By using a FACScan (Becton Dickinson, Heidelberg, Germany), equipped with the CELL Quest software, data were collected and analyzed. The results show the number of apoptotic cells reflected by the percentage of hypoploidy (subG1). In the end, the current apoptotic effects were calculated by subtracting the background apoptosis of the control cells from total apoptosis seen in the treated cells.

### Immunoblotting

After 48 h of incubation with different concentrations of WQF 044, Nalm6 cells were washed twice with PBS and lysed in buffer containing 10 mM Tris–HCl, pH 7.5, 300 mM NaCl, 1% Triton X-100, 2 mM MgCl_2_, 5 µM ethylenediamino tetra-acetic acid (EDTA), 1 µM pepstatin, 1 µM leupeptin, and 0.1 mM phenylmethyl-sulfonyl fluoride (PMSF). The same process was made with untreated RM82 and RM82SiHoVCR cells. By using the bicinchoninic acid assay from Pierce (Rockford, IL, USA), the protein concentration was determined and equal amounts of protein were separated by SDS–PAGE (Laemmli 1970; Smith 1985). The immunoblotting was performed as described (Wieder et al. 1994). The blocking of the membrane was made for 1 h in PBST (PBS, 0.05% Tween-20) containing BSA and incubated with different primary antibodies for 1 h. The anti-caspase-8, anti-caspase 9, anti-caspase-3, and anti-beta-actin from Sigma, Saint Louis, USA were used. After washing of the membrane with PBST, secondary antibody (anti-mouse IgG HRP from Bioscience, San Diego, USA and anti-rabbit IgG HRP from Promega, Minneapolis, USA) in PBST was applied for 1 h. After washing, the protein bands were detected using the ECL enhanced chemiluminescence system (Amersham Buchler, Braunschweig, Germany). The western blot quantification was done using GeneTools (Syngene).

### Measurement of the mitochondrial permeability transition

Nalm6 cells were treated with different concentrations of WQF 044 and incubated for 48 h at 37 °C. The cells were then centrifuged at 1500 rpm, 4 °C for 5 min. The JC-1 dye (5,5′,6,6′-tetrachloro-1,1′,3,3′-te-traethyl-benzimidazolyl-carbocyanin io-dide, Molecular Probes, Leiden, The Netherlands) was used on the cells to determine the mitochondrial permeability transition as described (Lambert et al. 1989; Reers et al. 1995). Most of the cells were resuspended in 500 µl phenol red free RPMI 1640 without supplements, and JC-1 was added to give a final concentration of 2,5 µg/µl. Control cells were left without JC-1. Thus, all cells were incubated for 30 min at 37 °C and moderately shaken. To collect the cells again, they were centrifuged at 1500 rpm for 5 min at 4 °C. 200 µl ice-cold PBS was used to wash cells. The flow cytometric determination of cells with decreased fluorescence was used to measure the mitochondrial permeability transition. The FACScan (Becton Dickinson, Heidelberg, Germany) was equipped with the CELL Quest software and analyzed the samples. Control cells with low ΔΨ_m_ were subtracted from the values observed in the treated cells. Data are given in percentage of cells with low ΔΨ_m_, which reflects the number of cells undergoing mitochondrial apoptosis.

### Gene expression analysis

The differential expression of multiple genes involved in the different apoptosis pathways was analyzed by using the apoptosis specific RT2 profiler (polymerase chain reaction) PCR expression arrays (SuperArray PAHS-012Z; SABiosciences Corporation, Frederick, MD, USA), according to the manufacturer’s instructions (Inohara et al. 1997). Nalm6 cells were incubated with 1 µM of WQF 044 for 16 h, RM82 and RM82SiHoVCR cells were left untreated. Then total RNA was extracted from the cells. RNAs were treated with Dnase I (2 U/µl) to eliminate possible genomic DNA contamination. The total RNA (700 ng/µl) was used as a template for the synthesis of a cDNA probe. It was then subjected to quantitative real-time PCR SuperArray analysis according to the manufacturer’s instructions using a LightCycler480 (Roche Diagnostics). The hybridization signals were normalized by the means of nine housekeeping genes. The results were analyzed by using the SuperArray Analyzer Software. Data are given as x-fold expression of the respective genes as compared with control (untreated Nalm6 or RM82 cells) cells incubated in vehicle-containing medium for 16 h.

### Isolation of healthy human leucocytes

10 ml RPMI 1640 medium was added to 50 ml blood of a healthy test person. Then, 4 ml of Ficoll (Saccharose-Epichlorhydrin-Copolymer) was pipetted in a 15-ml tube, followed by carefully adding 5 ml blood on the top. After 18 min of centrifugation at 657*g* (20 °C), the leucocytes were collected by slowly transferring them with a Pasteur pipette into a 45-ml tube. 20 ml of RPMI 1640 was added. This solution was centrifuged at 1500 rpm for 5 min. The cell count and viability were determined by CASY cell counter and analyzer system from Roche. Cells were seeded at a density of 3 × 10^5^ cells/ml. The following steps are identical to those in the measurement of DNA fragmentation as described above.

### Statistical analysis

Statistically significant values were compared using a one-tailed *t* test. *p* values were expressed with asterisk. *p* < 0.05 (*) was considered statistically significant, *p* < 0.01 (**) highly significant and *p* < 0.001 (***) extremely significant. If *p* > 0.05 is calculated, the result is not significant (n.s.).
